# ProCMD: a database and 3D web resource for protein C mutants

**DOI:** 10.1186/1471-2105-8-S1-S11

**Published:** 2007-03-08

**Authors:** Pasqualina D'Ursi, Francesca Marino, Andrea Caprera, Luciano Milanesi, Elena M Faioni, Ermanna Rovida

**Affiliations:** 1 Department of Science and Biomedical Technologies, University of Milano, Italy; 2 Institute of Biomedical Technologies, National Research Council, Segrate (Mi), Italy; 3 Hematology and Thrombosis Unit, DMCO- University of Milano and Az. Ospedaliera San Paolo, Italy

## Abstract

**Background:**

Activated Protein C (ProC) is an anticoagulant plasma serine protease which also plays an important role in controlling inflammation and cell proliferation. Several mutations of the gene are associated with phenotypic functional deficiency of protein C, and with the risk of developing venous thrombosis. Structure prediction and computational analysis of the mutants have proven to be a valuable aid in understanding the molecular aspects of clinical thrombophilia.

**Results:**

We have built a specialized relational database and a search tool for natural mutants of protein C. It contains 195 entries that include 182 missense and 13 stop mutations. A menu driven search engine allows the user to retrieve stored information for each variant, that include genetic as well as structural data and a multiple alignment highlighting the substituted position. Molecular models of variants can be visualized with interactive tools; PDB coordinates of the models are also available for further analysis. Furthermore, an automatic modelling interface allows the user to generate multiple alignments and 3D models of new variants.

**Conclusion:**

ProCMD is an up-to-date interactive mutant database that integrates phenotypical descriptions with functional and structural data obtained by computational approaches. It will be useful in the research and clinical fields to help elucidate the chain of events leading from a molecular defect to the related disease. It is available for academics at the URL .

## Background

Activated protein C (APC) is a vitamin K-dependent serine protease that plays a central role in the regulation of blood coagulation. It is the key component of an anticoagulant system that provides an essential mechanism in thrombosis prevention and in inflammatory response control.

APC binds to its cofactor protein S, and the complex inactivates the two cofactors involved in the clotting cascade, factors Va and factor VIIIa, leading to the efficient inhibition of the coagulation process. Furthermore APC has direct and indirect anti-inflammatory actions. It prevents leukocyte rolling, tissue factor exposure and tumour necrosis factor production by monocytes, thrombin-mediated inflammatory actions and apoptosis of endothelial cells [1]. The anti-thrombotic and anti-inflammatory actions of APC have been therapeutically exploited in severe sepsis [2].

Protein C, the zymogen of APC, is synthesized as a single chain precursor containing an amino-terminal leader sequence followed by a propeptide, which are cleaved upon secretion. It circulates in plasma mostly as a two-chain zymogen obtained by removal of the dipeptide Lys198 and Arg199 that results in the formation of two chains (light, 21 kDa and heavy, 41 kDa) linked by a disulphide bridge between Cys183 and Cys320. The zymogen is activated by thrombin through the proteolytic cleavage of a 12 amino acid peptide (Asp200-Arg211) [3]. The activation occurs on the endothelium of blood vessels by the thrombin-thrombomodulin complex [4] and this process is enhanced by the endothelial cell protein C receptor (EPCR) [5].

Protein C has a multi-domain structure: the light chain contains a γ-carboxyglutamic acid (Gla)-rich membrane binding domain and two epidermal growth factor (EGF)-like modules, while the heavy chain has the form of a typical trypsin-like serine protease domain [3].

The structure of the Gla-domainless APC has been solved by X-ray crystallography [6], while the structure of the Gla-domain is available in the complex with endothelial protein C receptor [7]. Protein C shares homologies with other vitamin K-dependent coagulation proteins as a result of a common evolutionary pathway.

Mutations on the gene have been found in patients with protein C deficiency (OMIM 176860), a disorder associated with development of purpura fulminans and severe recurrent thrombotic events in the homozygous form while, in the heterozygous form, it is responsible for an increased risk of venous thromboembolism in early adulthood [8,9].

Phenotypically, two distinct types of protein C deficiency are recognized: type I deficiency, the most common, is characterized by a parallel reduction in protein C concentration and function (measured in plasma by amidolytic or anticoagulant methods); type II deficiency is identified by normal or increased concentration and reduced function [10].

A large number of the mutations that contribute to the protein C deficiency fall in the structurally solved domains and thus are amenable to homology modelling and computational analysis.

Structural models can be useful in the research and clinical fields to elucidate how a mutation may interfere with enzymatic activity, ligand binding and cofactor interaction and relate the effect to patient phenotype.

In the last published database of mutations of the protein C gene [10,11], 161 different mutations (corresponding to 351 entries) are reported. Additional variants can be obtained from other sources (Human Gene Mutation Database [12,13] and the Swiss-Prot Variant Page [14,15]. However, to our knowledge, none of the available collections include data on the structural-functional interpretation of reported variants.

We describe here an updated, 3D-structure oriented database of protein C that associates clinical and phenotypical descriptions with functional and structural data obtained by computational approaches. It includes the description of 21 new variants that we have identified and analyzed in a previous work [16][17]. The database is integrated with an interactive search interface and with tools for structure visualization and mutant modelling.

## Construction and content

### Dataset

We collected a total of 195 naturally occurring mutations in the coding region of the protein C gene that include 182 missense and 13 stop mutations. Of these, a set of 21 variants were identified by our group through the screening of the protein C gene of 42 patients with a phenotypic functional deficiency of protein C [17]. The remaining portion of the dataset consists of missense and nonsense variants already reported, obtained from 3 different sources: the database of mutations of protein C gene [10,11], the Human Gene Mutation Database [12,13] and the Swiss-Prot Variant Page [14,15]. Additional variants, not included in the above databases, were obtained from literature. The entries were manually extracted and filtered to avoid duplicates.

General information associated with each entry was derived from literature sources. A multiple alignment obtained with CLUSTALW [18] using a set of orthologous and paralogous sequences was also associated with each entry.

Variants with substitutions in the structurally solved regions of the protein C, have been modelled starting from X-ray coordinates (PDB entry 1AUT). Molecular modelling of the 21 variants identified by our group, were achieved by residue replacement using InsightII (Accelrys INC., San Diego, Ca, USA). The lowest energy rotamer was chosen as the starting side chain position, followed by energy minimization calculation consisting of 500 steps of steepest descent keeping the backbone fixed, followed by 500 steps of conjugate gradient on the whole structure. A detailed computational analysis of these variants, such as electrostatic potential calculations, was formerly carried out and the results are also stored in the database.

All other variants were modelled using an automatic approach based on an adapted Python script of Modeller [19]. The script replaces the side chain of the mutated residue in the PDB file (1AUT) and optimizes the conformation by energy minimization and molecular dynamics. For each variant modelled, a set of 3D molecular representation were constructed using the programs PyMol (PyMOL Molecular Graphics System, DeLano Scientific, San Carlos, CA, USA) and MOLSCRIPT [20] to obtain fixed images and VRML (Virtual Reality Modelling Language) files. VRMLs can be viewed through a browser in a dynamic, interactive way using a player like CORTONA2 [21].

### Database and User Interface

The data are stored in a relational database managed by a MySQL Database Management System [22]. The database at the moment contains four tables: a "Mutations" table with all the single point mutation entries, a "Variants" table with clinical comments and, if available, literature data concerning each mutant; and two other tables with structural information about domains, chains, secondary structures, molecular modelling results and structure-function relationships.

We created a web based interface with the aim of helping users search for specific information, to browse the entire database or to visualize 2D and 3D images of the variants. The web site is also a source of documentation about protein C, and a point of access to external resources and databases related to protein C. This user interface has been built with PHP language scripts [23] on an Apache Web Server [24].

### Entry description

All entries in the database are labeled by an unique identifier and may include the following fields:

- sequence position of the mutated residues numbered according to:UniProtKB/Swiss-Prot entry P04070, Foster's codon numbering [25] and the chymotrypsin numbering used in PDB entry 1AUT when the substituted residue is included in the X-ray structure of protein C;

- wild type and mutated residues;

- gene localization;

- clinical or laboratory phenotype data as obtained from other database reports or from the literature;

- links to PUBMED database;

- cross-references to other databases reporting the mutation.

- There is also a structural information section that assigns the mutated residue to its specific chain, secondary structure and domain localization. A multiple alignment of homologous sequences, a 3D gallery of structural images and the PDB coordinates of the mutants are also present. Results of computational analysis are collected in a 3D-notes page that includes considerations on the physico-chemical properties of the mutant residue compared to the wild-type, (i.e. charge, hydrophobicity, solvent accessibility), a list of hydrogen bonds and hydrophobic interactions. Additional information resulting from further computational studies, such as electrostatic potential calculation, and the prediction of structural-functional effects of the mutation are associated with some entries.

### Utility and Discussion

The ProCMD database aims to provide a summary of the sequence and structure information on variants with substitutions in the coding region of the protein C gene. The database is interfaced with a fully interactive website, through which the user can retrieve entries of interest, find cross-references and visualize structural models with interactive tools.

**Figure 1:**
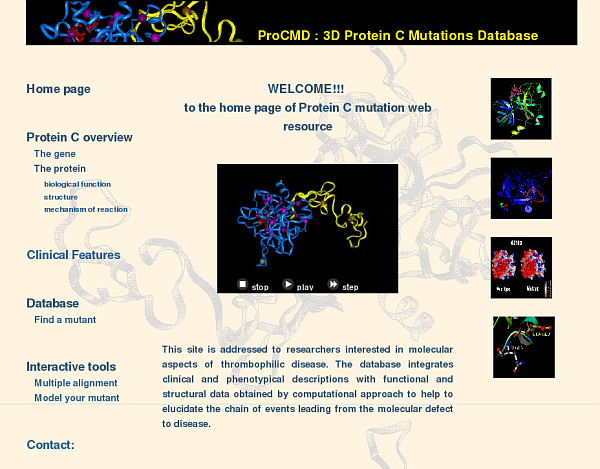
**The Home Page of the Database**. The database menu is on the left-hand side of the web page. It contains links to general information about gene, protein and clinical features of protein C deficiency, a link to the search in the database, links to mutations analysis tools, to authors correspondence, to a submission form for new mutations and to external web resources. Images are examples of structural details.

The home page of ProCMD web tool is shown in figure 1. Images and animations represent some examples of structural details of protein C variants included in the database. The menu on the left provides links to protein description pages that summarize literature data on protein structure and function and clinical features. It also provides a link to the database search interface and to modelling tools as described below.

### Data search and retrieval

A query page allows the user to retrieve entries by the position in the sequence of a mutated residue, by amino acidic substitution, and by domain localization. Results of the query are listed in a table showing the amino acidic substitution and the sequence position for each entry, and provide links to details pages summarizing all the associated data.

**Figure 2:**
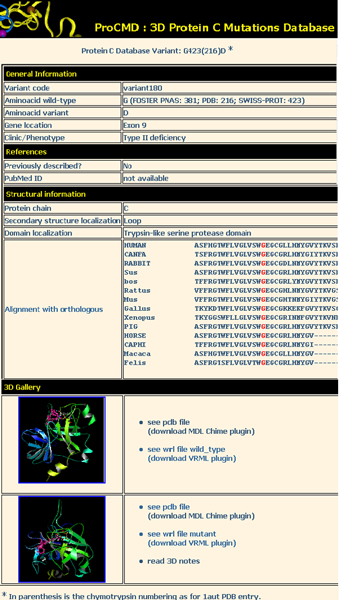
**Detailed view of an entry of the database**. The example refers to the variant G216D. The user may find general information about the variant, the available references and cross-references. A great importance is given to the structural aspects of the mutation. In the 3D gallery the user can access the PDB coordinates of the modelled mutant and the VRML format for visualization and a link to 3D notes.

An example of the output is shown in figure 2. A distinctive characteristics of ProCMD is that the mutation can be evaluated in the context of the structural features of protein C: information on secondary structure, and domain localization helps to predict whether a substitution can be tolerated in the protein structure or is likely to affect protein stability. Sequence alignments of orthologous and paralogous proteins are shown in the entry page with the residue of interest highlighted in red. This feature will help to evaluate the degree of conservation of the involved residue in the context of structurally and/or functionally relevant positions. Homology models of variants are available as PDB files and can be visualized with molecular graphics tools. In addition, images of the modelled mutant, mapping the substituted residue and the surrounding region, have been created and can be seen either in a static view or interactively through VRML. The coordinates and images of the wild type are also available to facilitate comparison. The VRML tool provides a ready view of mutation location and of functional residues on the 3D-structure and also displays interactions such as H-bonds, salt- and disulphide-bridges.

**Figure 3:**
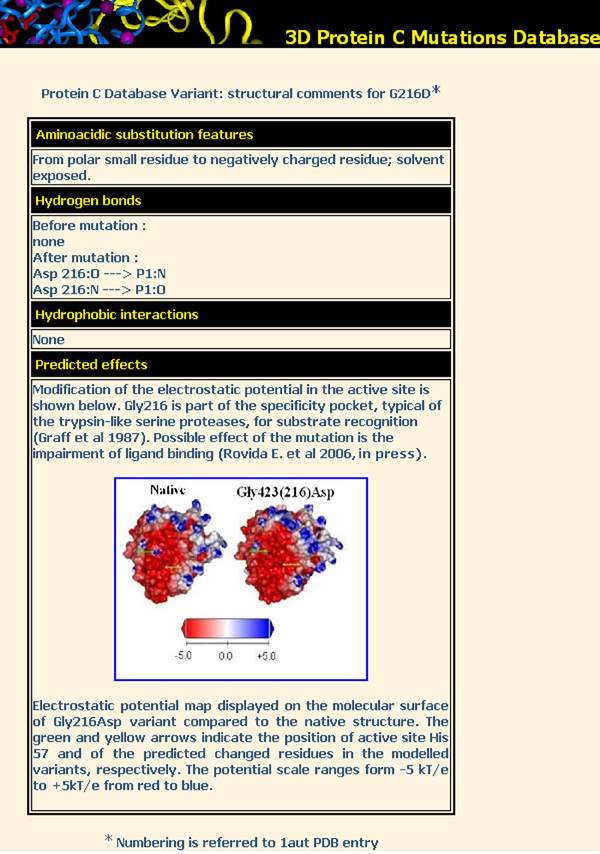
**A 3D-notes page**. The example refers to the variant G216D. Information on the physico-chemical properties of the mutant residue compared to wild-type (i.e. charge, hydrophobicity, solvent accessibility) is given, hydrogen bonds and hydrophobic interactions are listed. Additional data resulting from computational studies, such as electrostatic potential calculation, and the predicted effect of mutation are also reported.

Results of more detailed computational analysis and interpretation of the effect of mutation, when available, appear in a 3D-notes page with the corresponding images (figure 3).

Taken together, all the data related to each variant are useful to understand the relationship between the mutation and phenotype and help to elucidate the role of specific residues for protein function.

### Analysis tools for new mutations

For other user-defined missense mutants, not present in the database, the site provides tools for evaluating the residue conservation and for the homology modelling of the variant.

Selecting "Multiple alignments" from the home page (figure 1), the user can promptly visualize the residue of interest in the homologous alignment to have hints on its evolutionary conservation.

Molecular models can be obtained, if the residue falls in the 3D-structure, by the same automatic procedure based on the script of Modeller [19] used for entries preparation. Models can be visualized and 3D coordinates can be downloaded for further studies.

As the models are obtained in a completely automatic way, the user is cautioned about the possibility of having obtained non-accurate results. Careful inspection of the outcome is therefore recommended.

### Data submission

ProCMD features an online submission of mutation data. New mutations regarding the coding region of the protein C gene can be sent by filling out the fields on the online submission form. A text mail will be automatically generated by the server and sent to the authors after submission. The database curators verify the submitted data and will incorporate them after annotation according to the database format.

## Conclusion

This database provides a tool, complementary to other mutant collections of protein C, that is especially devoted to structural analysis and interpretation. A great effort has been put into the production of 3D-images associated with the molecular models and input files for interactive viewers which visualize the models in 3D space. The availability of structural models can be useful in the research and clinical fields both to elucidate how a mutation may interfere with enzymatic activity, ligand binding and cofactor interaction, and to relate the effect to patient phenotype. The present resource can be valuable to help predict the effect of a mutation, to clarify the role of specific residues in protein function and hopefully to give hints for the rational design of specific variants of protein C for therapeutic use.

## Availability and requirements

The database is maintained on the server of the Institute of Biomedical Technology -National Research Council (Segrate – MI, Italy) and is available at the following URL 

## Abbreviations

ProCMD:protein C mutation database, PDB:Protein Data Bank, APC: Activated Protein C, HGMD: Human Gene Mutation Database, VRML: Virtual Reality Modelling Language.

## Authors' contributions

PD conceived and designed the database and drafted the manuscript, FM carried out the data collection and annotation, implemented the SQL database and the web server pages and drafted the manuscript, AC structured the database and supervised the implementation, LM contributed to manuscript revision, EMF contributed to data communication and critically revised the manuscript, ER coordinated and supervised the project and prepared the manuscript. All authors read and approved the final manuscript
